# Antibiotic prescribing for acute respiratory tract infections in the United States outpatient setting

**DOI:** 10.1186/s12875-019-0980-1

**Published:** 2019-07-02

**Authors:** Amy L. Shaver, David M. Jacobs, Michael J. LaMonte, Katia Noyes

**Affiliations:** 1 Department of Epidemiology and Environmental Health, School of Public Health and Health Professions, University at Buffalo, 272 Farber Hall, South Campus, Buffalo, NY 14260 USA; 2 Department of Pharmacy Practice, School of Pharmacy and Pharmaceutical Sciences, University at Buffalo, Buffalo, NY USA

**Keywords:** Antibiotics, United States, General practice, Upper respiratory tract infection, outpatient

## Abstract

**Background:**

Acute respiratory tract infections (ARIs) are common in the outpatient setting. Although they are predominantly viral, antibiotics are often prescribed for the treatment of ARIs.

**Methods:**

Using the U.S. Medical Expenditure Panel Survey (MEPS; 2010–2015), we estimated the national prevalence and predictors of outpatient antibiotic prescribing for ARIs by provider type. We categorized the trends of antibiotic prescriptions (overall or broad-spectrum) for ARIs by provider type (physician and advanced practice provider [APP] which includes nurse practitioner [NP], and physician assistant [PA]). The outcome variable was defined as receipt of an antibiotic prescription during a consultation with a provider for an ARI (including outpatient clinic visit or doctor’s office visit).

**Results:**

There were 64,081,892 ARI antibiotic prescriptions written, with a decrease from 10.9 (2010) to 9.7 million (2015) during the study interval (*p* < 0.0001). Associations of patient- and provider-level variables with antibiotics prescription were examined using binary logistic regression. Blacks were more likely to receive antibiotics than whites (OR 1.51; 95% CI 1.25, 1.84; *p* < 0.001), and antibiotic prescription was more likely if the patient-provider race was concordant (OR 5.41; 95% CI 4.65, 6.29, *p* < 0.0001). Although the majority of patients with ARI were cared for by physicians, APPs were seeing an increasing number of ARI patients.

**Conclusions:**

Antibiotic prescribing for ARIs though declining, remains high. More research is needed to better understand the drivers of ARI antibiotic prescribing and to develop targeted interventions for both patients and providers.

**Electronic supplementary material:**

The online version of this article (10.1186/s12875-019-0980-1) contains supplementary material, which is available to authorized users.

## Background

Antibiotic resistance is a major public health concern, and its development is primarily due to the overuse, or inappropriate use, of antibiotics [1, 2]. Compounding the problem of antibiotic resistance is the fact that new antibiotics are not available quickly enough to mitigate antibiotic resistance at the population level [2, 3]. As concerns over antibiotic resistance increase, the demand for new antibiotics has also increased [4]. Antibiotic stewardship refers to “coordinated interventions designed to improve and measure the appropriate use of an agent by promoting the selection of the optimal drug regimen including dosing, duration of therapy, and route of administration [2]”. Within the policy set forth by the Society for Healthcare Epidemiology of America are recommendations including: that stewardship be required by regulation, that it be monitored in the ambulatory care (outpatient) setting, that providers and patients be educated on stewardship practices, and that stewardship research be completed [2]. Given the paucity of novel agents, stewardship efforts are increasing and their implementation encouraged in order to make best use of available antibiotics and limit the spread of antibiotic resistance [5]. A direct means of decreasing inappropriate antibiotic use would be to decrease inappropriate antibiotic prescribing. This could be done through the development of outpatient antibiotic stewardship programs including both patient and provider education on the dangers of inappropriate antibiotic prescribing.

Anywhere between 20 and 50% of the antibiotic prescribing in the United States outpatient setting is thought to be unnecessary; however, stewardship programs are not as prevalent in the outpatient setting as they are in the inpatient setting [2, 6–11]. Acute respiratory tract infections (ARIs) are common in the outpatient setting and usually do not require an antibiotic as they tend to be viral [12]. ARIs describe a range of conditions including acute bronchitis, nasopharyngitis, sinusitis, influenza, and often the symptoms associated with the common cold [12]. Patients with ARIs are often prescribed antibiotics, which can lead to increased antibiotic resistance [13]. Population-level surveillance may be preferred and viable method to systematically monitor antibiotic use for appropriateness [14, 15].

The prevalence of antibiotic prescriptions written by physician assistants and nurse practitioners have increased [11]. According to the Pew Charitable Trusts, the majority of antibiotics in the outpatient setting are prescribed by family-care physicians (41%), closely followed by physician assistants and nurse practitioners (23% combined) [16]. Knowing which providers are more likely to prescribe antibiotics as well as for what indication (such as ARIs) is critical for the development of effective and sustainable outpatient stewardship programs. Therefore, the objectives of this study were two-fold: (i) to provide an estimate of antibiotic prescribing for ARIs by provider group in the outpatient setting in the United States from 2010 to 2015 and (ii) to evaluate patient- and prescriber-level variables associated with prescribing antibiotics for ARIs.

## Methods

### Data source

The Medical Expenditure Panel Survey (MEPS) took its current form in 1996 and is administered by the Agency for Healthcare Research and Quality (AHRQ) [17]. MEPS participants are sampled from a subset of households who participated in the previous year’s National Health Interview Survey (NHIS). Respondents provide information over up to 2.5 years and five survey rounds (spaced 5–6 months apart); this information covers two years’ worth of a respondent’s information. MEPS oversamples from Hispanics, African Americans, Asians, and low-income individuals to increase the precision of generated estimates [17].

The current study utilized full-year consolidated household component files which contain expenditure and utilization data for the calendar year from several rounds of data collection. The household component includes the prescribed medicines file, which contains both medicine names and National Drug Codes (NDC) for all prescriptions. Prescription information, including NDC, is verified with the patient’s pharmacy. The prescribed medicines file also includes conditions associated with the medication, the start date of the medication, total expenditure, and sources of payment. Conditions are defined by truncated International Classification of Disease, 9th Revision, Clinical Modification (ICD-9-CM) codes, the truncated codes protect the privacy of survey participants. Trained professional coders complete the MEPS coding and determine the appropriate diagnosis code based on verbatim text from the participant. One respondent provides information for an entire household, and the consolidated full-year dataset, associated files, documentation, and codebooks are available through the AHRQ website [17].

### Study design and definitions

We analyzed outpatient ARI antibiotic prescribing categorized by provider from 2010 to 2015. Conditions of interest were those considered viral ARIs and were identified by ICD-9-CM and Clinical Classification System (CCS) codes. When a CCS code included a bacterial diagnosis, the more specific ICD-9-CM code was used. Events for inclusion were those with diagnosis codes for acute nasopharyngitis, ARI, acute bronchitis and bronchiolitis, laryngitis and tracheitis, influenza, and viral pneumonia.

ARI events were examined for antibiotic use. Antibiotic classes were identified using the NDC directory and generic names. Broad-spectrum antibiotics were defined based on the National Committee for Quality Assurance’s (NCQA) *Antibiotics of Concern* list and included quinolones, macrolides (azithromycin and clarithromycin), amoxicillin/clavulanate, ketolides (oral telithromycin), cephalosporins (2nd and 3rd generations), and clindamycin [11, 18–20]. Providers were categorized into two groups based on who the patient reported having seen during the healthcare visit. Those designated as medical doctor, doctor of osteopathy, or other medical specialty (surgeon, rheumatologist, etc.) were placed in the physician category. Nurse practitioners and physician assistants were collapsed into a single category, advanced practice provider (APP). As a provider could not be ascertained, 169,920,972 (26.9%) visits were excluded from the analysis.

### Variables of interest

Variables of interest were determined through literature review. Demographic variables included age, gender, race, geographic location, and income. Income was categorized as above and below the median for the sample (except in the regression analysis where it was kept as a continuous variable), and insurance coverage was categorized as private, public, or none. Race was described as a categorical variable including: White, Black, Asian, and Other. Geographic location was included as it can help bring to light regional variations in provider practice and its categorization is based on the US Census geographic regions: Northeast, South, Midwest, and West. Provider types were categorized as physician and APP as above. A race dyad was included to indicate whether the race of the provider was the same as the patient, with race determined based on respondents’ answers to survey questions. Number of comorbidities was included as a proxy of general health status. MEPS includes codes for fifteen distinct comorbidities (attention deficit hyperactivity disorder, angina, joint pain, high blood pressure, arthritis, emphysema, diabetes, elevated cholesterol, coronary heart disease, chronic bronchitis, cancer, asthma, history of stroke, history of heart attack, and other heart disease). A further patient characteristic included was an SF-12 indicator, the SF-12 being a population health measure and a suitable measure of self-reported health status in epidemiological studies (SF-12 is a 12-question short form) [21–23].

### Data and statistical analysis

Antibiotic prescription prevalence was defined as the percentage of ARI events wherein an antibiotic was prescribed; the prevalence was further expressed as the percent of ARI events wherein a broad-spectrum antibiotic was prescribed. A sensitivity analysis was conducted by comparing prevalence of antibiotic prescribing for ARI among the overall group and a new group wherein comorbidities that were considered likely to warrant antibiotic use including mastoiditis, otitis media, soft tissue infection, urinary tract infection, chronic obstructive pulmonary disease, human immunodeficiency virus/acquired immunodeficiency syndrome, cystic fibrosis, and diabetes mellitus were removed. As the two analyses showed no meaningful difference, those with the aforementioned comorbidities were retained for all analyses. (See Additional file 1.)

Multivariable binomial logistic regression was performed to determine factors associated with ARI antibiotic prescribing by provider type. The outcome of interest was receipt of an antibiotic prescription for ARI. The exposures of interest included multiple patient and provider characteristics including: patient age, patient sex, patient race, patient insurance coverage, number of patient comorbidities, patient income level, patient geographical location, and a patient-provider race dyad. As a further exploration of race, each homogenous group was compared against those visits where a concordant race dyad did not exist. The reference group for each exposure was determined based on literature review or lack of characteristic (in the case of the race dyad). Trend analyses year to year were performed utilizing a chi-square statistic. All statistical analyses were carried out utilizing the weighting as provided by AHRQ and using the survey procedures available in SAS version 9.4 (SAS Institute, Cary, NC).

## Results

### Antibiotic receipt categorized by demographics

During the study period (2010–2015) there were 461,647,174 visits to providers associated with a ARI diagnosis **(**Table 1**)**. Females were more frequently seen by a provider for a ARI than males, and therefore females were more frequently prescribed an antibiotic for ARI (62.42% vs. 37.58%). Those aged under 10 years accounted for the majority of visits (21.53%), and the percentage of ARI visits receiving an antibiotic were highest in the Southern region of the US (42.24%). As the number of comorbidities increased (a possible indication of poorer health), the number of antibiotics prescribed for a ARI decreased. Likewise, a higher SF-12 which indicates the patient believes they are in good health, was associated with an increased percentage of antibiotic receipt (above average: 39.05%, below average 25.06%). Physicians wrote 94.19% of antibiotic prescriptions for ARI visits compared to 5.81% written by advanced practice providers (nurse practitioners and physician assistants). Among whites seen for ARI, 48.9% saw a white provider whereas among Asian ARI visits, 39.7% saw an Asian provider and among blacks seen for ARI, 12.3% saw a black provider.

**Table 1:** Study population characteristics of patients with ARIs based on being prescribed an antibiotic^a^

Characteristic Total *n* = 461,647,174 (100%)	Prescribed antibiotic *n* = 67,974,312 (14.7%)	Not prescribed antibiotic *n* = 393,672,862 (85.3%)	*p* ^*c*^
Sex			0.0825
Male *n* = 181,521,291 (39.3%)	25,543,444 (37.58%)	155,977,847 (39.62%)	
Female *n* = 280,125,884 (60.7%)	42,430,868 (62.42%)	237,695,016 (60.38%)	
Age (years)			0.0647
Under 10 *n* = 110,656,003 (23.9%)	14,635,674 (21.53%)	96,020,329 (24.39%)	
10–19 *n* = 56,838,785 (12.3%)	7,772,409 (11.43%)	49,066,377 (12.46%)	
20–29 *n* = 37,182,206 (8.1%)	5,274,446 (7.76%)	31,907,759 (8.11%)	
30–39 *n* = 47,768,926 (10.3%)	7,550,521 (11.11%)	40,218,405 (10.22%)	
40–49 *n* = 51,518,968 (11.2%)	8,492,247 (12.49%)	43,026,721 (10.93%)	
50–59 *n* = 68,657,383 (14.9%))	10,492,186 (15.44%)	58,165,197 (14.78%)	
60–69 *n* = 55,017,935 (11.9)	8,304,438 (12.22%)	46,713,497 (11.87%)	
70–79 *n* = 22,470,007 (4.9%)	3,692,925 (5.43%)	18,777,082 (4.77%)	
≥ 80 n = 11,536,962 (2.5%)	1,759,465 (2.59%)	9,777,496 (2.48%)	
Race			< 0.0001
White *n* = 392,701,683 (85.1%)	60,176,049 (88.53%)	332,525,634 (84.47%)	
Black *n* = 34,685,841 (7.5%)	3,965,300 (5.83%)	30,720,541 (7.80%)	
Asian *n* = 15,916,805 (3.4%)	1,630,550 (2.40%)	14,286,255 (3.63%)	
Other n = 18,342,845 (4.0%)	2,202,413 (3.24%)	16,140,432 (4.10%)	
Region			0.0042
Northeast *n* = 76,084,919 (16.5%)	11,525,183 (16.96%)	64,559,736 (16.40%)	
Midwest *n* = 103,118,002 (22.3%)	17,331,387 (25.50%)	85,786,615 (21.79%)	
West *n* = 84,054,804 (18.2%)	10,403,926 (15.31%)	73,650,879 (18.71%)	
South *n* = 198,177,099 (42.9%)	28,713,816 (42.24%)	169,463,283 (43.05%)	
Family income			0.0413
$0 - $68,571 *n* = 231,258,378 (50.1%)	32,547,281 (47.88%)	198,711,097 (50.48%)	
≥ $68,571 *n* = 230,388,796 (49.9%)	35,427,031 (52.12%)	194,961,766 (49.52%)	
Insurance coverage			0.0030
Any private *n* = 343,417,418 (74.4%)	53,106,220 (78.13%)	290,311,198 (73.74%)	
Public only *n* = 100,535,778 (21.8%)	12,460,230 (18.33%)	88,075,548 (22.37%)	
Uninsured *n* = 17,693,978 (3.8%)	2,407,861 (3.54%)	15,286,117 (3.88%)	
Medicare Eligible			0.0530
Yes *n* = 57,709,393 (12.5%)	9,367,275 (13.78%)	48,342,118 (12.28%)	
No *n* = 403,937,782 (87.5%)	58,607,037 (86.22%)	345,330,745 (85.49%)	
Comorbidities			0.1114
0 *n* = 197,076,466 (42.7%)	27,548,817 (40.53%)	169,527,649 (43.06%)	
1 *n* = 91,760,462 (19.9%)	13,417,990 (19.74%)	78,342,471 (19.90%)	
2 *n* = 61,345,949 (13.3%)	9,941,271 (14.63%)	51,404,678 (13.06%)	
3 *n* = 50,259,383 (10.9%)	8,283,590 (12.19%)	41,975,793 (10.66%)	
4 n = 28,513,861 (6.2%)	4,156,770 (6.12%)	24,357,091 (6.19%)	
5 or more *n* = 32,691,054 (7.1%)	4,625,874 (6.81%)	28,065,180 (7.13%)	
SF-12 (only ≥ 18 years old)			0.0009
Below average *n* = 167,250,051 (36.2%)	17,033,112 (25.06%)	103,297,923 (26.24%)	
Average *n* = 21,250,631 (4.6%)	24,397,901 (35.89%)	154,427,749 (39.23%)	
Above average *n* = 162,490,490 (35.2%)	26,543,299 (39.05%)	135,947,191 (34.53%)	
Prescriber type			< 0.0001
APP n = 40,158,044 (8.7%)	3,950,115 (5.81%)	36,207,929 (9.20%)	
MD *n* = 421,489,131 (91.3%)	64,024,197 (94.19%)	357,464,934 (90.08%)	
Race same as provider			< 0.0001
Yes *n* = 202,777,280 (43.9%)	51,003,278 (75.03%)	151,774,002 (38.55%)	
No *n* = 258,869,894 (56.1%)	16,971,034 (24.97%)	241,898,861 (61.45%)	
White *n* = 192,129,340 (41.6%)^d^	49,216,309 (72.4%)	8,034,694 (2.0%)	
Black n = 4,258,487 (0.92%)^d^	783,968 (1.2%)	621,015 (0.16%)	
Asian n = 6,326,667 (1.4%)^d^	997,969 (1.5%)	961,236 (0.24%)	
Not concordant n = 258,869,894 (56.1%)^d^	16,971,034 (24.97%)	241,898,861 (61.45%)	

Abbreviations: *ARI* acute respiratory tract infection, *PA* physician assistant, *NP* nurse practitioner, *MD* physician, *SF-12* short form-12

^a^ All data presented as both weighted frequency (n) and proportions of patients (%)

^b^ Comorbidities include: attention deficit hyperactivity disorder, angina, joint pain, high blood pressure, arthritis, emphysema, diabetes, elevated cholesterol, coronary heart disease, chronic bronchitis, cancer, asthma, history of stroke, history of heart attack, and other heart disease

^c^ *p*-value represents results of χ2 for categorical values and t-test for continuous variables

^d^ Percentages do not sum to 100 as the ‘other’ category was too small to include

### ARI antibiotic receipt trends categorized by year and provider

**Fig. 1:**
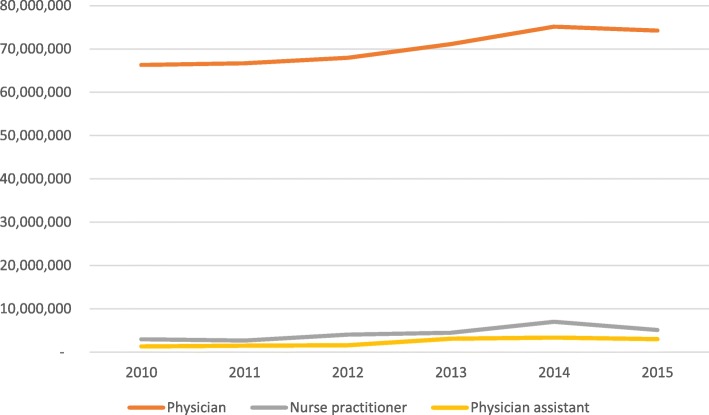
Prevalence of ARI categorized by provider type, 2010–2015

**Fig. 2:**
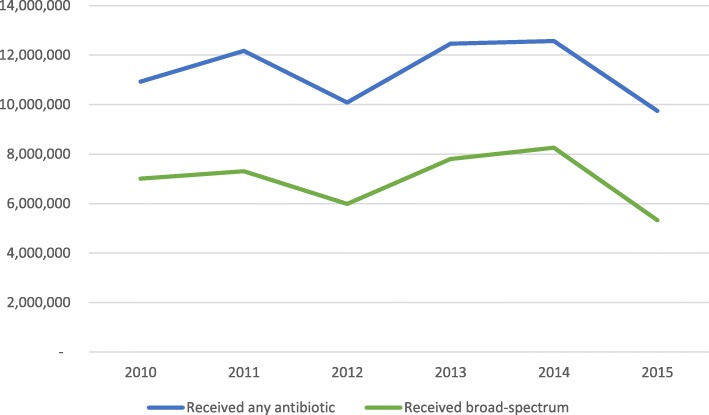
Prevalence of antibiotic type for ARI, 2010–2015

A total of 67,974,312 ARI antibiotic prescriptions were dispensed during the study period, with an approximately 10% decrease from 2010 to 2015 **(**Table 2**)**. The number of ARI visits increased during the study period from 70.6 million in 2010 to 82.4 million in 2015. Although a majority of ARI visits are managed by a physician that number is slowly decreasing as more ARI visits are managed by both nurse practitioners and physician assistants (Fig. 1). The percentage of ARI visits where an antibiotic was prescribed has decreased from 2010 (15.5%) to 2015 (11.8%) as has the percent of ARI visits where a broad-spectrum antibiotic was prescribed (Table 2, Fig. 2).

**Table 2:** Annual ARI prevalence categorized by year, type of provider seen, and antibiotic receipt^a^

Year	2010	2011	2012	2013	2014	2015
ARI Visits	70,564,592	70,866,608	73,615,067	78,723,720	85,523,707	82,353,481
Physician	66,303,919 (93.9%)	66,680,009 (94.1%)	67,972,755 (92.3%)	71,130,678 (90.4%)	75,152,677 (87.9%)	74,249,092 (90.2%)
Nurse practitioner	2,939,945 (4.2%)	2,665,883 (3.8%)	4,037,377 (5.5%)	4,477,586 (5.7%)	6,999,795 (8.2%)	5,094,616 (6.2%)
Physician assistant	1,320,728 (1.9%)	1,520,716 (2.2%)	1,604,935 2.2%)	3,115,456 (3.9%)	3,371,235 (3.9%)	3,009,773 (3.7%)
Received any antibiotic	10,927,504 (15.5%)	12,176,720 (17.2%)	10,087,197 (13.7%)	12,463,573 (15.8%)	12,570,746 (14.7%)	9,748,572 (11.8%)
Received broad-spectrum	7,013,417 (9.9%)	7,308,493 (10.3%)	5,988,583 (8.1%)	7,805,243 (9.9%)	8,260,365 (9.7%)	5,329,877 (6.5%)

Abbreviations: *ARI* acute respiratory tract infection

^a^ Data presented as number of ARI visits, and number of visits (%)

Broad-spectrum includes: quinolones, macrolides (azithromycin and clarithromycin), amoxicillin/clavulanate, ketolides (oral telithromycin), cephalosporins (2nd and 3rd generations), and clindamycin

The percentage of ARI visits where an antibiotic was prescribed categorized by the type of provider seen is presented in Table 3. Among ARI visits managed by a physician 15.2% received an antibiotic, with 9.4% of visits receiving a broad-spectrum antibiotic. Among those visits managed by an advanced practice provider (a category made up of nurse practitioners and physician assistants) 9.8% received an antibiotic and 5.4% received a broad-spectrum antibiotic.

**Table 3:** ARI antibiotics categorized by provider seen from 2010 to 2015

Provider type	Physician^a^	Advanced practice provider^b^
Antibiotic prescribed	64,024,197 (15.2%)	3,950,115 (9.8%)
Broad-spectrum prescribed	39,535,243 (9.4%)	2,170,736 (5.4%)

Abbreviation: *ARI* acute respiratory tract infection

^a^ Data presented as number of physician ARI visits where an antibiotic was prescribed followed by percent of physician visits where an antibiotic was prescribed

^b^ Data presented as number of advanced practice provider (APP) ARI visits where an antibiotic was prescribed followed by percent of APP visits where an antibiotic was prescribed

Broad-spectrum includes: quinolones, macrolides (azithromycin and clarithromycin), amoxicillin/clavulanate, ketolides (oral telithromycin), cephalosporins (2nd and 3rd generations), and clindamycin

### Factors associated with ARI antibiotic receipt

Factors associated with antibiotic prescription during a ARI visit are presented in Table 4. Black patients were more likely than their white counterparts to receive an antibiotic for a ARI diagnosis (OR 1.51; 95% CI 1.25, 1.84). Although the South saw more cases of ARI those in the Midwest had a 18% greater odds of antibiotic receipt for ARI than those in the South (OR 1.18; 95% CI 1.01, 1.38). Those with an above average SF-12 score were 21% more likely to have received an antibiotic for ARI (OR 1.21; 95% CI 1.03, 1.42). As comorbidity increased, odds of antibiotic receipt decreased insignificantly (OR 0.97; 95% CI 0.93, 1.01). Those ARI events managed by physicians as compared to those managed by advanced practice providers (nurse practitioners and physician assistants) were at an increased odds of antibiotic receipt (OR 1.36; 95% CI 1.13, 1.65). The odds of ARI antibiotic receipt were increased when the race of the patient matched the race of the provider (OR 5.41; 95% CI 4.65, 6.29). Odds varied depending on whether the concordant race was White, Black or Asian compared to odds in non-concordant pairs (OR 3.98; 95% CI 1.96, 8.08; OR 2.61; 95% CI 1.21, 5.65; OR 2.23; 95% CI 1.06, 4.69, respectively).

**Table 4:** Factors associated with receipt of an antibiotic during a ARI visit^a,b^

Characteristic	OR^a,b^	95% CI	*p* ^*e*^
Age (years), continuous	0.99	0.99, 1.00	0.0785
Sex			
Female	1.08	0.97, 1.19	0.1494
Male (ref)			
Race			
Black	1.51	1.25, 1.84	< 0.001
Asian	0.72	0.56, 0.93	0.0118
Other	2.24	1.60, 3.13	< 0.0001
White (ref)			
Comorbidities^c^, continuous	0.97	0.93, 1.01	0.1492
Region			
Northeast	1.03	0.87, 1.21	0.7421
Midwest	1.18	1.01, 1.38	0.0411
West	0.93	0.80, 1.09	0.3775
South (ref)			
Income, continuous	1.00	1.00, 1.00	0.4597
Insurance coverage			
Any Private	1.05	0.76, 1.44	0.7756
Any Public	0.85	0.61, 1.17	0.3167
Uninsured (ref)			
Medicare			
Yes	1.09	0.90, 1.32	0.3868
No (ref)			
SF-12			
Above average	1.21	1.03, 1.42	0.0190
Below average	1.14	0.94, 1.37	0.1813
Average (ref)			
Provider type			
Physician	1.36	1.13, 1.65	0.0016
APP (ref)			
Concordant race^d^			
Yes	5.41	4.65, 6.29	< 0.0001
No (ref)			
White	3.98	1.96, 8.08	0.0002
Black	2.61	1.21, 5.65	0.0153
Asian	2.23	1.06, 4.69	0.0353
Not concordant (ref)			

Abbreviations: *ARI* acute respiratory tract infection, *OR* odds ratio, *CI* confidence interval, *APP* advanced practice provider

^a^ The odds compare those ARI visits where antibiotics were prescribed to those ARI visits where no antibiotic was prescribed

^b^ Each estimate is adjusted for all other variables in the table

^c^ Comorbidities include: attention deficit hyperactivity disorder, angina, joint pain, high blood pressure, arthritis, emphysema, diabetes, elevated cholesterol, coronary heart disease, chronic bronchitis, cancer, asthma, history of stroke, history of heart attack, and other heart disease

^d^ Concordant race refers to the patient and provider being of the same race as reported by the patient

^e^ p-value represents a test for significance of the odds ratio

The results of a sensitivity analysis, not shown, comparing prevalence of antibiotic prescribing for ARI among the overall group and a new group wherein comorbidities that were considered likely to warrant antibiotic use including mastoiditis, otitis media, soft tissue infection, urinary tract infection, chronic obstructive pulmonary disease, human immunodeficiency virus/acquired immunodeficiency syndrome, cystic fibrosis, and diabetes mellitus were removed, indicated no meaningful difference between the groups in regards to population characteristics nor proportion of antibiotic prescribing. (See Additional file 1.)

## Discussion

The purpose of this study was to determine the prevalence of antibiotic prescribing categorized by provider type, as well as to examine if any factors were associated with ARI antibiotic prescribing. This study found that most antibiotics for ARI are prescribed by physicians. An increasing number of ARI cases are seen by APPs even though the vast majority of ARIs are seen by physicians. The authors found that racial disparities exist in antibiotic prescribing for ARI. Interestingly, the study found that when the race of the patient and provider was concordant the patient was more likely to be prescribed an antibiotic for ARI. It is heartening to see that antibiotic prescribing for ARI is decreasing, but it still remains at a high level.

Although physicians saw the majority of ARI cases over the study period, the percentage of ARI cases being seen by physicians decreased concurrently with an increase in the proportion of cases seen by nurse practitioners and physician assistants. This trend may be due to a number of related factors. Although the overall number of physicians has increased to serve the population, there has also been an increase in the number of physicians seeking specialization [24–26]. The physician to patient ratio in primary care settings has remained close to 50 primary care physicians per 100,000 persons from 1980 through 2010 regardless of federal government incentives to increase the number of community-based primary care physicians [24, 27]. It has also been suggested that the relatively lower salary and a medical educational culture that fosters specialization would need to change in order to recruit and train enough primary care physicians to face the projected shortage [27–31]. Alongside a decrease in primary care physicians, there has been an upsurge in the number of retail clinics and urgent care centers following changes in non-physician practitioner scope of practice regulations [32]. The quality of care at retail clinics has been shown to be comparable to physician offices but at lower cost for the treatment of otitis media, pharyngitis, and urinary tract infections [33]. The combination of lower cost and convenience of appointment may account for the increase in ARI cases managed by APPs.

Antibiotic prescribing for ARIs remained relatively stable among physicians over the study period when considered as a percentage of ARI visits. Granted, a higher percentage of physician visits saw an antibiotic prescribed and a higher percentage of broad-spectrum antibiotic. However, this may be due to physicians seeing more complex patients at a later stage in the disease process. Among nurse practitioners and physician assistants, antibiotic prescribing for ARIs increased. The increase in antibiotic prescribing among APPs is consistent with previous findings. In a cross-sectional study using a nationally representative database covering 2005 to 2010, antibiotic prescribing rates decreased for physicians but increased by 3.2 and 3.4% among physician assistants and nurse practitioners, respectively [11]. Similarly, there has been an increase in broad-spectrum antibiotic prescribing for ARIs. Our findings show a significant increase in broad-spectrum prescribing among physician assistants, with a slight increase among nurse practitioners. Suda et al. reported a 15% increase in broad-spectrum prescribing among APPs and a decrease among physicians [11]. Lee et al. performed an analysis of outpatient antibiotic prescribing using the MEPS database from 2000 to 2010 and found that broad-spectrum prescribing doubled over that period [19]. Utilizing the National Ambulatory Medical Survey and the National Hospital Ambulatory Medical Care Survey, Roumie et al. showed that non-physician clinicians prescribe antibiotics more frequently in situations that are deemed inappropriate regardless of practice setting [34]. Antibiotic prescribing in the outpatient setting is critical to combatting antibiotic resistance [35].

There existed evidence of racial disparity in ARI antibiotic prescription receipt, with blacks more likely to receive an antibiotic than their white counterparts. Gerber et al. showed a decreased likelihood of antibiotic receipt among black children and, when prescribed an antibiotic, it was less likely to be broad-spectrum [36]. Goyal et al. described similar findings among non-Hispanic black children [37]. The current study examined both provider and patient race, which may account for the differing results. Race concordance between provider and patient was strongly associated with antibiotic receipt for an ARI. Though the strength of the association differed each concordant race pair was in the same direction away from the null. Other studies focusing on race examined the race of the patient but did not include the race of the provider [36]. The patient-provider relationship represents a complex interplay between the perceived wants of the patient or guardian and the perception by providers that a patient who does not ‘get what they want’ will not return to their practice [36]. It has previously been shown that although patients may sometimes desire an antibiotic when it is not warranted, the ability of a provider to accurately interpret a patient’s desire for an antibiotic prescription and to predict this desire is flawed [38]. Mangione-Smith et al. showed that when a provider gave extra counseling regarding the lack of need for an antibiotic, most patients left a provider office satisfied with their experience [38]. Further research is warranted to examine the role race plays in the decision to prescribe an antibiotic for both the provider and patient.

This study found that those in the South had a higher prevalence of ARI office visits. Concurrently the patients seen in the Midwest were more likely to receive an antibiotic at a ARI office visit. Government figures indicate that the highest rates of antibiotic prescribing is in the South followed by the Midwest, however this is for all antibiotics and not only those for ARI [39]. This study also found that as number of comorbidities increased the odds of antibiotic decreased, though the finding was not significant. This could be due to different use of the healthcare system. The study also found that as a person’s perception of their health status improved the odds of antibiotic receipt for ARI increased. In other words, those who are in good health with a low number of comorbidities may only go to a provider when they are suffering from more ‘everyday’ complaints such as the common cold.

Viral infections do not benefit from treatment with an antibiotic. Prescribing an unwarranted medication increases the incidence of adverse drug events and leads to increased antibiotic resistance [40, 41]. Judicious use of antibiotics is currently a focus of inpatient antibiotic stewardship programs, and although there is a move towards stewardship in the outpatient setting, efforts need to be expanded and reinforced to decrease antibiotic prescribing and related expenditures [8, 42–45]. Expansion of such efforts can help to further decrease potentially unwarranted antibiotic prescribing. Patients need to be educated that an antibiotic is not always a necessity and, in some cases, may in fact cause harm. Clinicians need to reinforce the concept of appropriate antibiotic use with their patients and attempt to decrease their prescribing rate, particularly for viral indications [46]. A key component of antibiotic stewardship programs is continuing education and this should be provided to all clinicians [47]. This study has extended previous work by considering different factors that may be at play in the patient-provider relationship. Specifically, this study considered both patient and provider characteristics as factors associated with ARI antibiotic prescribing.

This study has several limitations. First, the study depended on ICD-9-CM and CCS codes from patient self-report, which could lead to misclassification of diagnosis leading to a misinterpretation of either a patient’s comorbidities or of ARI. ARIs are typically viral in origin and previous studies removed visits from analysis where it was believed that patient comorbidities could more reasonably dictate an antibiotic prescription. Initial removal of these ARI visits from the current study population was done so as to clarify the results; in other words, by removing cases where it may be appropriate to find an antibiotic prescribed, we can rest more assured that where we find an antibiotic prescribed it is more likely to be inappropriate. It was determined that removal of those persons did not lead to a significant difference in the proportion of ARI visits where an antibiotic was prescribed and as such the study was conducted on all ARI visits where a provider could be determined. Second, within multi-provider practices, the clinician who signed off on a prescription may not be the provider who initially prescribed it. The MEPS Household component reports who the patient saw during their visit and concluded that the provider seen by the patient was the one who wrote the prescription. Regardless, we believe that our estimates provide a useful baseline of antibiotic prescribing for a common viral illness. A further limitation is the use of income as a marker of socioeconomic status versus the more robust marker years of education. This was done due to the inconsistency of the years of education variable across the study years in MEPS. The analysis was based on complete data of both the exposure and the outcome which caused the removal of approximately 27% of ARI cases for missing a provider which may have caused inaccuracy in the estimate. As an observational study, it is possible that estimates were inaccurate due to the presence of unmeasured confounders.

## Conclusions

In conclusion, ARI antibiotic prescribing has decreased over the study period namely in the final year of the study. Though it is possible the difference seen in prescribing may not be a true reduction, only future study will tell if there are other factors at work that could have accounted for the differences observed. Racial differences in antibiotic prescribing were seen, especially when the race of the patient matched the race of the provider. Further research is necessary to determine the role that race plays in the patient-provider relationship with regards to antibiotic prescribing. Further, targeted stewardship programs should be developed to help decrease the potentially inappropriate prescribing habits of all providers.

## Additional file

Additional file 1: **Appendix 1.** Sensitivity Analysis. **Table S1.** Population characteristics. (DOCX 20 kb)

## Data Availability

The datasets generated and/or analyzed during the current study are available in the AHRQ MEPS repository, https://meps.ahrq.gov/mepsweb/data_stats/download_data_files.jsp.

## Abbreviations

AHRQ: Agency for Healthcare Research and Quality
APP: Advanced Practice Provider
ARI: Acute Respiratory Tract Infection
CCS: Clinical Classification System
CI: Confidence Interval
ICD-9-CM: International Classification of Disease, 9th Revision, Clinical Modification
MD: Medical Doctor, physician
MEPS: Medical Expenditure Panel Survey
NCQA: National Committee for Quality Assurance
NDC: National Drug Code
NHIS: National Health Interview Survey
NP: Nurse Practitioner
OR: Odds Ratio
PA: Physician Assistant
SF: Short form
