# Supplementary material for: Antibiotic prescribing for acute respiratory tract infections in the United States outpatient setting
Source: BMC Fam Pract. 2019 Jul 2;20:91. doi: 10.1186/s12875-019-0980-1 (PMC6607511; doi:10.1186/s12875-019-0980-1)
Supplement: Supplementary file 1 — Appendix 1. Sensitivity Analysis. Table S1. Population characteristics. (DOCX 20 kb) [file 12875_2019_980_MOESM1_ESM.docx]

Appendix 1: Sensitivity Analysis

Table S1: Population characteristics

| **Characteristic Total n =** 461,647,174 **(100%)** | **Population with no indication for exclusion**  **n =** 317,471,475 | **Population with indication for exclusion**  **n =** 144,175,699 |
| --- | --- | --- |
| **Sex** |  |  |
| Male n= 181,521,291 | 130,666,608 | 50,854,683 |
| Female n= 280,125,884 | 186,804,867 | 93,321,017 |
| **Age (years)** |  |  |
| Under 10 n= 110,656,003 | 72,826,562 | 37,829,440 |
| 10-19 n=56,838,785 | 46,098,130 | 10,740,655 |
| 20-29 n=37,182,206 | 30,389,508 | 6,792,697 |
| 30-39 n= 47,768,926 | 37,700,804 | 10,068,122 |
| 40-49 n=51,518,968 | 39,564,633 | 11,954,335 |
| 50-59 n=68,657,383 | 39,664,077 | 28,993,306 |
| 60-69 n= 55,017,935 | 32,335,009 | 22,682,927 |
| 70-79 n=22,470,007 | 13,161,397 | 9,308,611 |
| ≥80 n=11,536,962 | 5,731,355 | 5,805,607 |
| **Race** |  |  |
| White n = 392,701,683 | 268,982,559 | 123,719,123 |
| Black n = 34,685,841 | 24,374,940 | 10,310,901 |
| Asian n = 15,916,805 | 12,648,688 | 3,268,117 |
| Other n = 18,342,845 | 11,465,288 | 6,877,558 |
| **Region** |  |  |
| Northeast n = 76,084,919 | 53,375,561 | 22,709,358 |
| Midwest n = 103,118,002 | 67,444,865 | 35,673,137 |
| West n = 84,054,804 | 59,527,268 | 24,527,538 |
| South n = 198,177,099 | 136,918,836 | 61,258,263 |
| **Family income** |  |  |
| $0 - $68,571 n = 231,258,378 | 154,625,938 | 76,632,440 |
| ≥$68,571 n = 230,388,796 | 162,845,537 | 67,543,259 |
| **Insurance coverage** |  |  |
| Any private n = 343,417,418 | 241,929,570 | 101,487,848 |
| Public only n = 100,535,778 | 64,508,936 | 36,026,843 |
| Uninsured n = 17,693,978 | 11,032,970 | 6,661,009 |
| **Medicare Eligible** |  |  |
| Yes n= 57,709,393 | 33,290,105 | 24,419,288 |
| No n= 403,937,782 | 284,181,370 | 119,756,411 |
| **Comorbidities** |  |  |
| 0 n = 197,076,466 | 150,290,714 | 46,785,752 |
| 1 n = 91,760,462 | 70,301,536 | 21,458,926 |
| 2 n = 61,345,949 | 44,465,145 | 16,880,804 |
| 3 n = 50,259,383 | 29,951,453 | 20,307,929 |
| 4 n = 28,513,861 | 14,028,284 | 14,485,577 |
| 5 or more n = 32,691,054 | 8,434,343 | 24,256,712 |

| **Characteristic Total n =** 461,647,174 **(100%)** | **Population with no indication for exclusion**  **n =** 317,471,475 | **Population with indication for exclusion**  **n =** 144,175,699 |
| --- | --- | --- |
| **SF-12** |  |  |
| Below average n = 167,250,051 | 106,111,739 | 61,138,312 |
| Average n = 21,250,631 | 15,878,997 | 5,371,634 |
| Above average n = 162,490,490 | 122,654,177 | 39,836,313 |
| **Prescriber type** |  |  |
| MD n = 421,489,131 | 290,083,304 | 4,255,280 |
| NP n = 26,215,201 | 17,700,608 | 8,514,593 |
| PA n = 13,942,842 | 9,687,563 | 131,405,827 |
| **Race same as provider** |  |  |
| Yes n= 202,777,280 | 139,182,999 | 63,594,281 |
| No n= 258,869,894 | 178,288,476 | 80,581,418 |
| **Received Antibiotic for URTI** |  |  |
| Yes n= 67,974,312 | 47,963,643 | 20,010,669 |
| No n= 393,672,862 | 269,507,832 | 124,165,030 |
| **Received Broad Antibiotic for URTI** |  |  |
| Yes n= 41,705,978 | 28,770,760 | 12,935,218 |
| No n= 419,941,196 | 288,700,715 | 131,240,481 |

Logistic Regression Results

| Characteristic | OR^a^ | OR^b^ | 95% CI^a^ | 95% CI^b^ |
| --- | --- | --- | --- | --- |
| Sex |  |  |  |  |
| Female | 1.078 | 1.071 | 0.973, 1.193 | 0.952, 1.206 |
| Male (ref) |  |  |  |  |
| Age, continuous | 0.996 | 0.995 | 0.992, 1.000 | 0.990, 1.000 |
| Race |  |  |  |  |
| Black | 1.511 | 1.461 | 1.245, 1.835 | 1.164, 1.833 |
| Asian | 0.722 | 0.704 | 0.561, 0.930 | 0.536, 0.924 |
| Other | 2.237 | 2.218 | 1.597, 3.133 | 1.565, 3.143 |
| White (ref) |  |  |  |  |
| Race same as provider |  |  |  |  |
| Yes | 5.409 | 5.560 | 4.653, 6.288 | 4.696, 6.583 |
| No (ref) |  |  |  |  |
| Region |  |  |  |  |
| Northeast | 1.028 | 1.009 | 0.872, 1.211 | 0.837, 1.217 |
| Midwest | 1.178 | 1.106 | 1.007, 1.379 | 0.937, 1.304 |
| West | 0.931 | 0.912 | 0.795, 1.091 | 0.772, 1.077 |
| South (ref) |  |  |  |  |
| Income |  |  |  |  |
| Above median | 0.958 | 0.910 | 0.863, 1.064 | 0.807, 1.025 |
| Below median (ref) |  |  |  |  |
| Ins coverage |  |  |  |  |
| Any Private | 1.047 | 0.949 | 0.761, 1.440 | 0.687, 1.311 |
| Any Public | 0.847 | 0.752 | 0.612, 1.173 | 0.532, 1.063 |
| Uninsured (ref) |  |  |  |  |
| Medicare |  |  |  |  |
| Yes | 1.090 | 1.029 | 0.896, 1.324 | 0.810, 1.309 |
| No (ref) |  |  |  |  |
| SF |  |  |  |  |
| Above average | 1.211 | 1.220 | 1.032, 1.420 | 1.021, 1.458 |
| Below average | 1.136 | 1.147 | 0.942, 1.370 | 0.939, 1.401 |
| Average |  |  |  |  |
| Comorbidities, continuous | 0.968 | 0.994 | 0.927, 1.012 | 0.942, 1.050 |
| Provider type (ref=NP-PA) |  |  |  |  |
| MD or DO | 1.364 | 1.403 | 1.126, 1.651 | 1.151, 1.711 |
| PA or NP (ref) |  |  |  |  |
| ^a^ Represents the results of a logistic regression using the full population  ^b^ Represents the results of a logistic regression excluding those with previously noted comorbidities | | | | |
